# Evaluation of Three Candidate Live-Attenuated *Salmonella enterica* Serovar Typhimurium Vaccines to Prevent Non-Typhoidal *Salmonella* Infection in an Infant Mouse Model

**DOI:** 10.3390/vaccines11101562

**Published:** 2023-10-04

**Authors:** Khandra T. Sears, Shamima Nasrin, Scott M. Baliban, Danielle N. Council, Marcela F. Pasetti, Sharon M. Tennant

**Affiliations:** 1 Center for Vaccine Development and Global Health, University of Maryland School of Medicine, Baltimore, MD 21201, USA; ksears@som.umaryland.edu (K.T.S.); sbaliban@som.umaryland.edu (S.M.B.); mpasetti@som.umaryland.edu (M.F.P.); 2 Department of Medicine, University of Maryland School of Medicine, Baltimore, MD 21201, USA; 3 Department of Pediatrics, University of Maryland School of Medicine, Baltimore, MD 21201, USA

**Keywords:** *Salmonella*, gastroenteritis, oral vaccine, nontyphoidal

## Abstract

Nontyphoidal *Salmonella enterica* (NTS) is a leading cause of foodborne illness worldwide, including in the United States, where infants show the highest incidence amongst all age groups. *S. enterica* serovar Typhimurium is one of the most frequently isolated serovars from NTS infections. We have developed several candidate live-attenuated *S.* Typhimurium vaccines to prevent NTS infection. The goal of the current study was to assess three live *S.* Typhimurium vaccine strains (CVD 1921, CVD 1921 ∆*htrA* and CVD 1926, which have two, three and four gene deletions, respectively) with various levels of reactogenicity and immunogenicity in infant BALB/c mice to predict how they would perform following peroral immunization of infants. We first tested intranasal immunization of 14-day-old mice with three doses delivered at 1-week intervals and evaluated antibody responses and protection against lethal infection with wild-type *S.* Typhimurium. The vaccines were administered to 14-day-old mice via the peroral route at 1- or 2-week intervals and to 28-day-old mice at 2-week intervals. The three vaccine strains were immunogenic following intranasal immunization of infant mice with vaccine efficacies of 80% (CVD 1921), 63% (CVD 1921 ∆*htrA*) and 31% (CVD 1926). In contrast, peroral immunization of 14-day-old mice yielded much poorer protection against lethal infection and only immunization of 28-day-old mice at 2-week intervals showed similar protective capacity as intranasal administration (CVD 1921: 83%, CVD 1921 ∆*htrA:* 43% and CVD 1926: 58%). CVD 1921 was consistently more protective than both CVD 1921 ∆*htrA* and CVD 1926, regardless of the route of vaccination, immunization schedule and age of mice. Anti-LPS serum IgG responses were similar between the three strains and did not correlate with protection. Due to previously observed reactogenicity of CVD 1921, CVD 1921 ∆*htrA* and CVD 1926 are our preferred vaccines, but these data show that further improvements would need to be made to achieve suitable protection in young infants when using peroral immunization.

## 1. Introduction

Enteric pathogens are a leading cause of infection worldwide [1]. Among the many pathogens that cause diarrheal disease, nontyphoidal *Salmonella* (NTS) is a common cause of acute gastroenteritis [1,2]. NTS is estimated to cause over 90 million infections and over 100,000 deaths globally every year [2]. In the United States alone, NTS causes approximately 1 million infections and thousands of hospitalizations annually [3,4]. Many serovars are circulating within the United States, and the *Salmonella enterica* serovar Typhimurium is among the five most frequently detected serovars [5]. Importantly, young children show the highest incidence of NTS infection; in 2016, infants (<12 months of age) had an incidence of 110.80 *Salmonella* infections per 100,000 for females and 108.81 per 100,000 for males, in contrast to an average incidence amongst all age groups of 14.65 and 13.25 per 100,000 for females and males, respectively [6].

Protective immunity against NTS infection includes local and systemic antibodies and cell-mediated responses [7,8]. Immunization at the site of infection, the gut mucosa in this context, via oral delivery elicits mucosal as well as systemic immunity, including humoral and cell-mediated immunity [8,9]. Hence, oral immunization against enteric pathogens is appealing because live-attenuated organisms engender immunity through the natural route of infection. In addition, oral vaccines offer an inexpensive and easy-to-administer platform for protection against gastrointestinal pathogens. They eliminate the need for needles and syringes and minimize the risk of handling materials contaminated with bloodborne pathogens. However, intrinsic host defenses, which include low pH, antimicrobial peptides and innate immune cells, may decrease the effectiveness of live oral vaccines. Additionally, in infants and young children, components in maternal milk may interfere with the viability of live vaccine strains and the infant’s immune response [10,11].

Infants can develop humoral and cell-mediated responses to vaccines delivered to mucosal sites [11]. The safety and efficacy of oral vaccines administered to infants and young children have also been established, as well as their value in reducing morbidity and mortality due to enteric infections. For example, the live oral rotavirus vaccines have successfully reduced the incidence of acute gastroenteritis due to rotavirus globally, with an estimated 2.6% decrease in mortality in children under 5 years between 2000 and 2016 [12,13]. Sublingual immunization against rotavirus has resulted in reduced circulation of group A virus strains and has had the added effect of reducing hospitalizations due to gastroenteritis in unvaccinated older children and adults [14,15]. Oral poliovirus vaccines (OPV), perhaps the most well-known live-attenuated oral vaccines, elicit protective humoral IgG and mucosal IgA antibody responses [11,16]. The oral live-attenuated *S.* Typhi vaccine, Ty21a (Vivotif), is delivered in a three- or four-dose regimen and reduced the incidence of typhoid fever in children and adults [17,18]. Therefore, there is ample evidence that live oral vaccines can elicit robust immune responses in young infants.

There are no licensed vaccines against NTS, and there has been limited assessment of candidate NTS vaccines in infant mice. There is a single study in which a live-vector *S.* Typhimurium vaccine was evaluated in infant mice [19]. The recombinant attenuated *Salmonella* vaccine (RASV) strain expressing heterologous antigens from *Streptococcus pneumoniae* was shown to be safe when administered to pregnant dams, neonates and infant mice via the intranasal and oral routes. Pregnant mice developed *S. pneumoniae* PspA-specific mucosal and systemic antibody responses, and infants were subsequently protected from lethal *S. pneumoniae* challenge. While systemic antibody responses to *S.* Typhimurium LPS were detected, protection against NTS infection was not determined.

Our strategy to develop NTS vaccines has included mutations in a combination of metabolic pathways and virulence factors to achieve a balance between safety and immunogenicity [20]. Using *S.* Typhimurium strain I77, a clinical isolate belonging to sequence type 19 (ST19) and the most prevalent ST globally [21], we have engineered multiple vaccine candidates. CVD 1921 (*S.* Typhimurium I77 Δ*guaBA* Δ*clpP*) is a guanine auxotroph that requires exogenous guanine to replicate. The double-knockout mutant is hyperflagellated due to the deletion of *clpP* [22]. Since excessive flagellin could result in inflammation due to activation of the inflammasome, we assessed pro-inflammatory cytokine gene expression in gut organoids and observed decreased expression of IL-1β compared to the parental strain, I77 [23], indicating that CVD 1921 is less reactogenic than I77. Mice immunized with CVD 1921 were highly protected (≥80% vaccine efficacy) against lethal challenge with the parent wild-type strain *S.* Typhimurium I77 [22]. However, CVD 1921 was associated with a low rate of vaccinemia in mice, although it was well tolerated in SIV-infected rhesus macaques [22,24]. Subsequent deletion of two additional genes, *pipA* and *htrA*, to generate CVD 1926 (I77 ∆*guaBA* ∆*clpP* ∆*pipA* ∆*htrA*) resulted in a better-tolerated vaccine. However, this vaccine strain only elicited 60% vaccine efficacy against lethal infection in mice [25]. CVD 1926 was able to protect rhesus macaques against *S.* Typhimurium moderate-to-severe gastroenteritis [23]. We have also shown that CVD 1926 was able to elicit robust T-cell responses in mice [26]. Altogether, we have demonstrated the safety and/or protective efficacy of orally delivered live-attenuated *S.* Typhimurium vaccines in adult animal models; however, to our knowledge, live oral NTS vaccines have not been assessed in infant mouse models of NTS infection.

The goal of this study was to assess three candidate *S.* Typhimurium vaccine strains—CVD 1921, CVD 1921 Δ*htrA* and CVD 1926, which represent a range of reactogenicity and immunogenicity—in infant mice. Although CVD 1926 is our preferred vaccine (least reactogenic), we hypothesized that it would not be sufficiently immunogenic in infant mice and hence also tested CVD 1921 and CVD 1921 ∆*htrA*. Strains deficient in *htrA* have reduced survival in macrophages and show reduced colonization of deep tissues compared to *aroA* mutants but still afford protection from wild-type challenge in mouse infection models [27,28]. We hypothesized that the deletion of *htrA* would not over-attenuate CVD 1921 but reduce its reactogenicity while maintaining its immunogenicity and protective efficacy. We investigated the systemic and mucosal responses elicited by these vaccines following delivery via different mucosal routes and at different dosing intervals in infant mice. We assessed their immunogenicity and protective efficacy against lethal *S.* Typhimurium challenge to determine the optimal immunization route and schedule for infants, i.e., the target population for these vaccines.

## 2. Materials and Methods

### 2.1. Bacterial Strains and Culture Conditions

The bacterial strains and plasmids used in this study are listed in Table 1. All *Salmonella* strains were maintained in animal-product-free Hy-Soy (HS) medium (10 g/L soytone (Teknova, Hollister, CA, USA), 5 g/L Hy-yeast (Kerry BioScience, Beloit, WI, USA) and 5 g/L sodium chloride (AmericanBio, Natick, MA, USA)) at 37 °C. Agar (AmericanBio) was added when necessary at 15 g/L. Medium was supplemented with guanine (0.005% (*w*/*v*) final; Sigma-Aldrich, St. Louis, MO, USA) for mutants harboring Δ*guaBA* deletions. Antibiotic (carbenicillin (Corning, Glendale, AZ, USA) or kanamycin (50 µg/mL final; Sigma-Aldrich)) was added when necessary. For immunization studies, vaccine strains were streaked onto HS medium containing guanine and grown for 18–20 h at 37 °C. Bacterial colonies were then resuspended in sterile phosphate-buffered saline (PBS) to a concentration of 10^8^ to 10^9^ colony-forming units (CFU) per 10 µL for infant mice and 100 µL for adult mice.

### 2.2. Construction of CVD 1921 ΔhtrA

CVD 1921 Δ*htrA* was constructed by deleting the *htrA* gene from the chromosome of CVD 1921 using lambda red-mediated homologous recombination [33]. A construct consisting of DNA upstream and downstream of *htrA* fused to a kanamycin resistance gene was generated by overlapping PCR. DNA upstream of *htrA* was amplified by PCR using primers htrAupF (5′ GGTACCTTCAATCAGGCGTTAC 3′) and htrAupR (5′ GTGTTTCAATCTCGATTAACAGGTAACGC 3′). DNA downstream of *htrA* was amplified using primers htrAdnF (5′ TCACCTTTGTCCCCCTTCCGCCATGGAAG 3′) and htrAdnR (5′ ATATTTACGCAGGTGCTCTGGT 3′). The kanamycin resistance gene from pKD13 was amplified using primers htrA-kanF (5′ TACCTGTTAATCGAGATTGAAACACGTGTAGGCTGGAGCTGCTTC 3′) and htrA-kanR (5′ TCCATGGCGGAAGGGGGACAAAGGTGACTGTCAAACATGAGAATTAA 3′). The three PCR products were combined by overlapping PCR and electroporated into CVD 1921 expressing lambda red recombinase as described previously [33]. The kanamycin resistance gene was removed following recombination at FRT sites that flank the gene. The gene deletion was confirmed by PCR and sequencing; sequencing primers included htrAseqF (5′ GAACGATATTCGCCGCAAGG 3′), htrAseqR (5′ GAGTACCACCATCTCGGTGAGAGAA 3′), and hrtAseqF2 (5′ CCAGAAACTTTATTCCGGAACTTCG 3′). Construction and characterization of CVD 1921 and CVD 1926 have previously been described [22,23].

### 2.3. Generation of Bioluminescent S. Typhimurium Strains

Bioluminescent *S.* Typhimurium vaccine strains were constructed by introducing pCM17 carrying the *luxCDABE* operon into vaccine strains CVD 1921, CVD 1921 Δ*htrA* and CVD 1926 by electroporation [34]. Bioluminescent colonies were detected using a ChemiDoc^TM^ MP imaging instrument with Image Lab 5.1 software (BioRad Laboratories, Hercules, CA, USA) and by measuring bioluminescence using a luminometer.

### 2.4. In Vivo Bioluminescence Imaging of S. Typhimurium Vaccines in Infant and Adult Mice

Fourteen-day-old (infant) and six- to eight-week-old (adult) BALB/c mice (*n* = 5) were immunized by peroral gavage with one dose (10^9^ CFU) of CVD 1921 or CVD 1921 ∆*htrA* or CVD 1926 strains containing plasmid-encoded *luxCDABE* or PBS. Mice were anesthetized and imaged at 2, 24, 48 and 65 h post-immunization (hpi) for 1 min with medium binning in a Xenogen IVIS 200 imaging system (Spectral Instruments Imaging, Tucson, AZ, USA). In vivo luminescence was calculated by generating a Region of Interest (ROI) circle using Living Image 4.5.2 software. Total photon flux (photons s^−1^) was used for all calculations and expressed as arbitrary units (AU). Animals were re-imaged at indicated time-points.

### 2.5. Mouse Immunization and Protection against Challenge

All animal procedures were approved by the Institutional Animal Care and Use Committee of the University of Maryland School of Medicine (protocol number 0619004). Adult (6- to 8-week-old) female and male BALB/c mice were obtained from Charles River Laboratories (Wilmington, MA, USA). Infant BALB/c mice were bred in our animal facility. Mice were weaned at 21 days of age. Mice had access to food and water at all times (i.e., they were not fasted prior to any procedures).

Groups of 6- to 8-week-old adult (*n* = 15 per group) BALB/c mice were given three doses of 10^9^ CFU in 100 µL of PBS by peroral gavage at 1-week or 4-week intervals. Two or four weeks after the last immunization, adult mice were challenged perorally with 2 × 10^6^ CFU (100 × LD_50_) wild-type (WT) *S.* Typhimurium I77. Viable counts were performed to confirm the number of bacteria administered to the mice. Control mice (*n* = 15) received 100 μL of PBS. Mice were monitored for morbidity and mortality for up to 35 days after challenge. Any mouse that lost more than 20% of its body weight as compared to its weight at the time of infection or that showed signs of extreme morbidity (e.g., shallow breathing or hunched posture) was euthanized and scored as a death.

To assess intranasal immunization of infant mice, groups of 14-day-old mice (*n* = 15 per group) were given three doses of 10^8^ to 10^9^ CFU of CVD 1921 or CVD 1921 ∆*htrA* or CVD 1926 in 10 µL of PBS at 1-week intervals. Two weeks after the last immunization, mice were orally challenged with 2–3 × 10^6^ CFU (100 × LD_50_) WT I77 in 100 µL PBS and monitored as described above. To assess peroral immunization of infant mice, 14-day-old mice (*n* = 15 per group) were given three doses of 10^8^ to 10^9^ CFU of each vaccine suspended in 10 µL PBS at 1-week and 2-week intervals. Mice were challenged with 2–3 × 10^6^ CFU WT *S.* Typhimurium I77 two weeks after the third dose of immunization and monitored for up to 35 days. To assess the efficacy of the vaccines post-weaning but before adulthood, 28-day-old mice were immunized with three doses of 10^9^ CFU in 50–100 µL of PBS by peroral gavage at 2-week intervals. Mice were challenged two weeks after the final dose of immunization and monitored as above.

### 2.6. Measurement of Serum and Fecal Antibodies

Blood was collected from each mouse prior to each immunization and challenge to determine the levels of serum IgG antibodies against *S.* Typhimurium core- and O-polysaccharide (COPS). COPS was harvested and purified from strain CVD 1925 (pSEC10-*wzzB*) as previously described [35]. For perorally immunized mice, feces were collected the day before challenge and resuspended to a concentration of 100 mg/mL in PBS containing 1% sodium azide and protease inhibitors to assess levels of anti-COPS fecal IgA antibodies. Antibody titers were determined by enzyme-linked immunosorbent assay (ELISA) according to the protocol described previously [36]. Briefly, medium-binding 96-well microtiter plates (Grenier Bio-One, Monroe, NC, USA) were coated with 100 μL/well of *S.* Typhimurium COPS antigen at a concentration of 5 μg/mL. COPS was diluted in phosphate-buffered saline (PBS, pH 7.4), and plates were incubated for 3 h at 37 °C. After incubation, plates were washed 6 times with 0.05% Tween 20 in PBS (PBST) and blocked with 250 μL/well of 10% non-fat dry milk (NFDM, RPI, Mt. Prospect, IL, USA) in PBS overnight at 4 °C. Individual mouse serum or fecal samples were diluted in 10% NFDM in PBST and tested in duplicate wells. Plates were incubated at 37 °C for 1 h and, at the end of incubation, washed 6 times with PBST. Horseradish peroxidase (HRP)-conjugated goat anti-mouse IgG or goat anti-mouse IgA secondary antibodies (Bio-Rad Laboratories, Carlsbad, CA, USA) were used according to the manufacturer’s instructions. Antibody binding was detected using the TMB Microwell Peroxidase Substrate system (KPL, Gaithersburg, MD, USA). Endpoint titers were calculated as the inverse of the serum dilutions that produced an absorbance value at 450 nm of 0.2 above the blank.

### 2.7. Statistical Analyses

For in vivo bioluminescence imaging, adult and infant mice were compared using an unpaired *t*-test (two-tailed). Vaccine efficacy data were analyzed using the log rank test. Differences in ELISA titers were analyzed using a two-tailed Mann–Whitney test. For all analyses, a *p*-value of ≤0.05 was considered significant.

## 3. Results

### 3.1. Immunogenicity and Vaccine Efficacy of S. Typhimurium Vaccines in Adult BALB/c Mice

**Figure 1:**
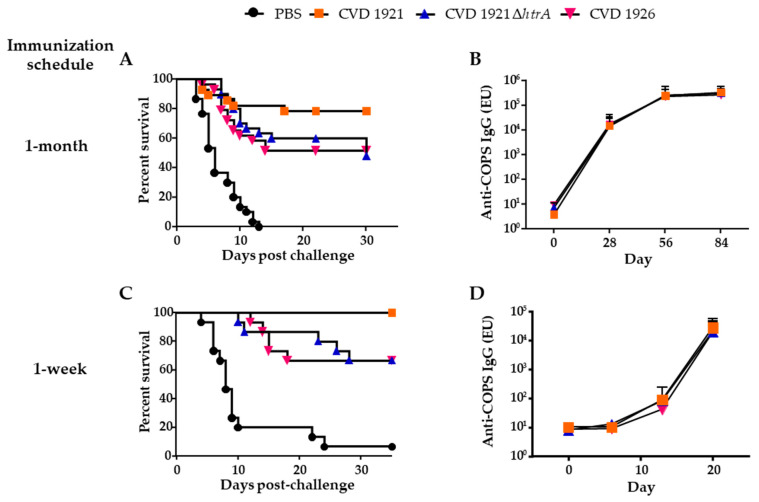
Immunogenicity and protective efficacy of *S.* Typhimurium vaccines CVD 1921, CVD 1921 ∆*htrA* and CVD 1926 in adult mice. Six- to eight-week-old BALB/c mice (n = 28 to 30) were immunized orally with 10^9^ CFU of vaccines or PBS three times at 4-week intervals (**A**,**B**) or at 1-week intervals (**C**,**D**) then orally challenged with 100 × LD_50_ *S.* Typhimurium strain I77 one month following the last immunization (the LD_50_ of I77 is 2 × 10^4^ CFU in adult BALB/c mice, which is similar to the common laboratory strain SL1344, which has reported LD_50′_s of 4 × 10^4^ CFU and 1.2 × 10^5^ CFU) [37,38]. Mice were monitored for 30 days after challenge to assess protection after lethal challenge (**A**,**C**) and serum anti-COPS IgG titers in response to each immunization and prior to challenge (**B**,**D**). Points indicate the mean ± standard deviation from all mice.

Peroral immunization of adult female 6- to 8-week-old mice with CVD 1921, CVD 1921 ∆*htrA*, CVD 1926 or PBS three times at 4-week intervals elicited robust serum antibody responses and protected mice from lethal challenge (Figure 1A,B). We also assessed a shortened immunization schedule in which the vaccines were administered 1 week apart and observed high vaccine efficacy (VE) for CVD 1921 and anti-COPS IgG titers (Figure 1C,D). Approximately one-third of the mice in each group seroconverted (≥four-fold rise in anti-COPS IgG titer) after the second dose (CVD 1921: 5/15 (33.3%), CVD 1921 ∆*htrA*: 4/15 (26.7%) and CVD 1926: 5/15 (33.3%)); following the third dose, seroconversion was observed in nearly all mice (CVD 1921: 15/15 (100%), CVD 1921 ∆*htrA*: 14/15 (93.3%) and CVD 1926: 15/15 (100%)). Importantly, mice immunized with the three vaccines using both dosing intervals were protected against lethal challenge (Table 2).

### 3.2. Immunogenicity and Vaccine Efficacy of S. Typhimurium Vaccines in Infant BALB/c Mice Following Intranasal Administration

**Figure 2:**
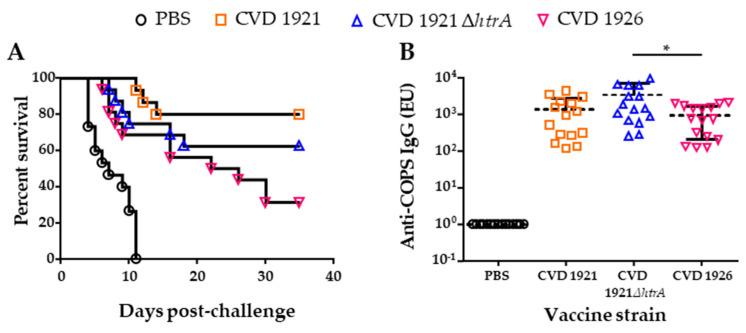
Survival following challenge and immunogenicity of infant mice immunized with live-attenuated *S.* Typhimurium vaccines intranasally. (**A**) Survival of 14-day-old mice immunized i.n. with 3 doses of CVD 1921, CVD 1921 ∆*htrA*, CVD 1926 or PBS and subsequently challenged with 100 × LD_50_ WT I77. (**B**) Anti-COPS serum IgG titers following 3 doses of indicated vaccines. Geometric mean titer represented by dashed line. Each point represents an individual mouse. * *p* ≤ 0.01 (Mann–Whitney test).

We delivered 10^9^ CFU of CVD 1921, CVD 1921 ∆*htrA* or CVD 1926, or PBS to 14-day-old BALB/c mice three times intranasally (i.n.) at one-week intervals. All vaccines were well tolerated, and no vaccine-induced mortality was observed. CVD 1921 and CVD 1921 ∆*htrA* demonstrated VEs of 80% and 63%, respectively, while the VE of CVD 1926 was 31% (Figure 2A, Table 3). High seroconversion rates for anti-COPS serum IgG were observed after the second dose of each vaccine strain (CVD 1921: 11/15 (73.3%), CVD 1921 ∆*htrA*: 12/16 (75.0%) and CVD 1926: 9/16 (56.3%)); all mice, except one in the CVD 1926 group, seroconverted prior to challenge. Overall, anti-COPS serum IgG titers after three doses of vaccine were similar among the three vaccine groups, although a significant difference between titers of CVD 1921 ∆*htrA-* compared to CVD 1926-immunized mice was detected (Figure 2B; *p* ≤ 0.01, Mann–Whitney test).

### 3.3. In Vivo Clearance of S. Typhimurium Vaccines in Infant and Adult BALB/c Mice Following Peroral Administration

**Figure 3:**
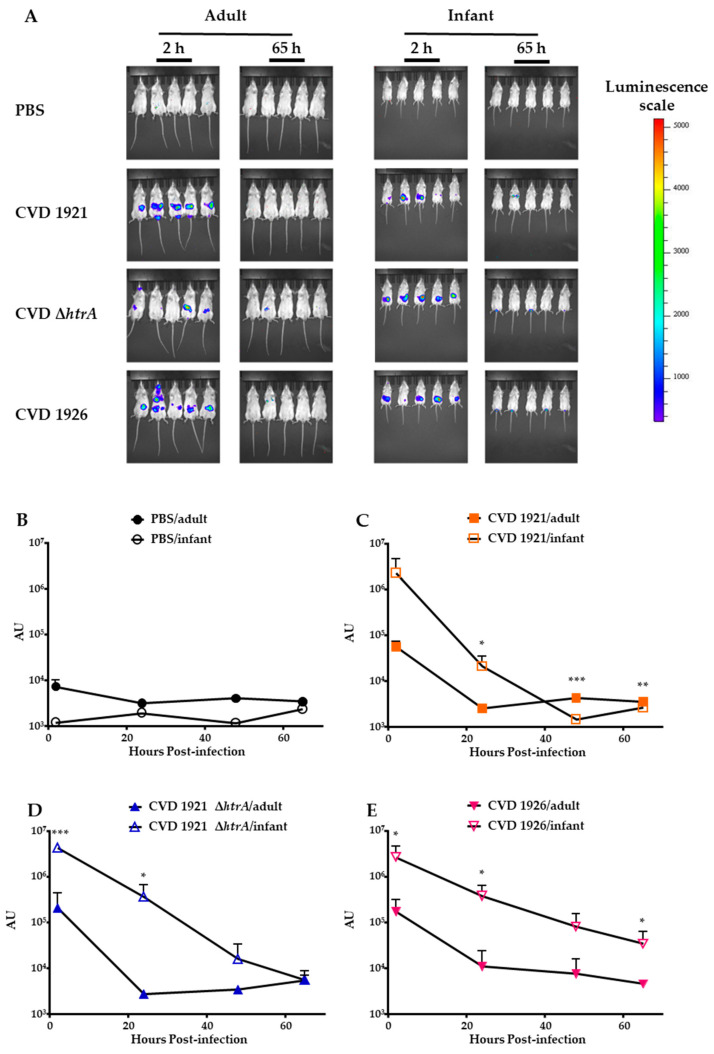
In vivo survival of *S.* Typhimurium vaccine strains in infant and adult mice. Six- to eight-week-old adult and infant BALB/c mice were PBS (sham)-immunized or immunized with bioluminescent versions of CVD 1921, CVD 1921 ∆*htrA* and CVD 1926. Mice were imaged for 1 min at 2, 24, 48 and 65 h post-infection to monitor bacterial clearance. (**A**) Images at 2 h and 65 h for adult and infant mice. (**B**–**E**) Total photon flux (photons s^−1^) was determined, and luminescence was expressed as arbitrary units (AU) for each time-point. Asterisks indicate significant differences in mean AU between infant and adult mice at the indicated times. * *p* ≤ 0.05, ** *p* ≤ 0.01, *** *p* ≤ 0.001 (unpaired *t*-test, two-tailed). Symbols represent the mean ± standard deviation.

We engineered bioluminescent versions of each vaccine strain to assess survival of the vaccine strains in vivo. Adult and infant mice were perorally administered luminescent versions of CVD 1921, CVD 1921 ∆*htrA* or CVD 1926. Control mice receiving PBS alone did not exhibit changes in luminescence over time (Figure 3A,B). The highest levels of luminescence in mice receiving vaccine strains in both age groups were captured at 2 hpi (Figure 3A,C–E). Significantly higher levels of luminescence were detected in immunized infant mice compared to adults at 24 h. At 65 hpi, AU levels between infants and adults receiving the vaccine strains were similar to levels in control mice.

### 3.4. Effect of Age and Immunization Schedule on Immunogenicity and Protection Following Peroral Administration

**Figure 4:**
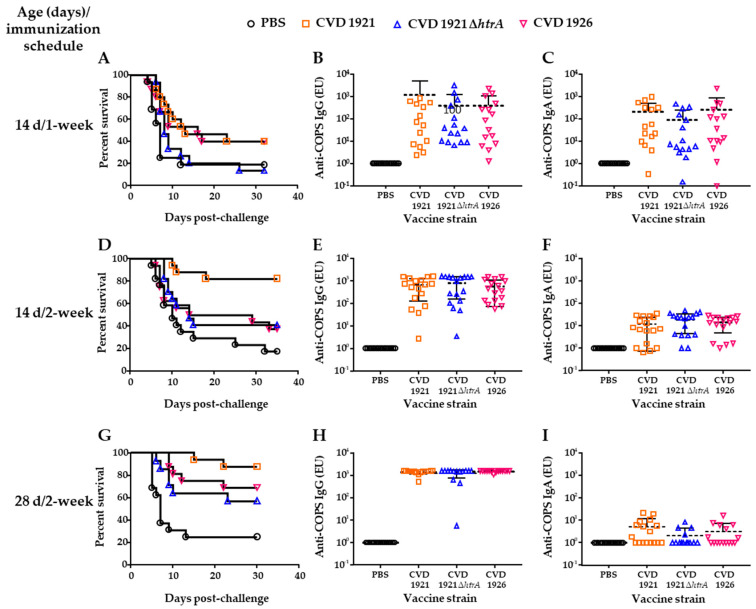
Mortality and immunogenicity of infant and young mice immunized with live-attenuated *S.* Typhimurium vaccines. Young mice at different ages were immunized p.o. with 3 doses of CVD 1921, CVD 1921 ∆*htrA*, CVD 1926 or PBS at 1- or 2-week intervals and subsequently challenged with WT *S.* Typhimurium I77. (**A**–**C**) 14-day-old mice at 1-week intervals; (**D**–**F**) 14-day-old mice at 2-week intervals; (**G**–**I**) 28-day-old mice at 2-week intervals. Leftmost panels (**A**,**D**,**G**) show survival curves, center panels (**B**,**E**,**H**) show anti-COPS serum IgG titers and right panels (**C**,**F**,**I**) show anti-COPS fecal IgA titers after three vaccine doses and prior to challenge. Geometric mean titer represented by dashed line. Each point represents an individual mouse.

We evaluated immunogenicity and protection of our candidate strains in 14-day-old infant mice immunized perorally. First, we immunized mice p.o. three times, with 1 week between each immunization, and challenged them with WT *S.* Typhimurium I77. Mice immunized with CVD 1921, CVD 1921 ∆*htrA* or CVD 1926 exhibited poor vaccine efficacy, and attack rates were not significantly different from the PBS control group (VE = 34%, 1.5% and 26%, respectively, Figure 4A and Table 4). Anti-COPS IgG seroconversion rates were low after the second vaccination (CVD 1921: 0/15 (0%), CVD 1921 ∆*htrA*: 1/15 (6.7%), and CVD 1926: 2/15 (13.3%)), and only two-thirds of mice in each group had seroconverted prior to challenge (CVD 1921: 10/15 (66.7%), CVD 1921 ∆*htrA*: 10/15 (66.7%), and CVD 1926: 10/15 (66.7%)). No significant differences in the geometric mean titer (GMT) in COPS-specific serum IgG and fecal IgA (Figure 4B,C) were observed between the vaccine groups, although responses were quite variable.

We next assessed an extended dosing schedule in which vaccines were delivered at 2-week intervals (i.e., 14, 28 and 42 days of age). VE for CVD 1921 was 79%; however, VEs for CVD 1921 ∆*htrA* and CVD 1926 were not significant and lower at 29% and 24%, respectively (Figure 4D and Table 4). No significant differences were observed in serum IgG COPS-specific antibody titers between the vaccines (Figure 4E). More than half of the mice in each group showed seroconversion for anti-COPS IgG after the second dose (CVD 1921: 10/17 (58.8%), CVD 1921 ∆*htrA*: 12/17 (70.6%) and CVD 1926: 11/16 (68.7%)); prior to challenge, seroconversion rates were 15/17 (88.2%) for CVD 1921, 16/17 (94.1%) for CVD 1921 ∆*htrA* and 16/16 (100%) for CVD 1926. There were no significant differences between vaccines in terms of fecal IgA titers (Figure 4F).

Finally, we investigated the impact of immunizing mice post-weaning at 28 days old. Peroral immunization of 28-day-old mice with three doses 2 weeks apart generally improved survival for mice immunized with both CVD 1921 ∆*htrA* and CVD 1926; while the VE for CVD 1921 ∆*htrA* was not statistically significant (VE = 43%, *p* = 0.1349), the VE for CVD 1926 was 58% (*p* = 0.0320; Figure 4G and Table 4). The VE for CVD 1921 was 83%. Prior to challenge, anti-COPS IgG seroconversion rates were 16/17 (94.1%) for CVD 1921, 14/15 (93.3%) for CVD 1921 ∆*htrA* and 16/16 (100%) for CVD 1926. There were no significant differences between vaccines in terms of anti-COPS serum IgG or fecal IgA titers (Figure 4H,I).

## 4. Discussion

In our continued development of live-attenuated NTS vaccines, we have assessed three *S.* Typhimurium vaccine candidates under different parameters in infant models of infection. We first confirmed that a shortened vaccination schedule (1-week intervals vs. 1-month intervals) in adult mice did not reduce VE before assessing the protective efficacy of these vaccine strains in 14-day-old mice. We initially immunized infant mice i.n. under the premise that maternal milk ingested by infant mice could reduce the viability of the vaccine strains and blunt immune responses. All three vaccines showed significant vaccine efficacy in infant mice, albeit generally at lower levels than observed in adults using the 1-week interval schedule. We used bioluminescent strains to address the question of vaccine strain survival in vivo and observed that, overall, the strains were cleared from infant and adult mice with similar kinetics. Finally, we investigated how the timing of peroral vaccinations and the age of initial dosing impacted efficacy. None of the candidate vaccines was protective in infant mice when doses were delivered perorally at 1-week intervals; however, the efficacy of CVD 1921 was restored by extending the interval to 2 weeks. Vaccine efficacies were also higher when delivered to mice post-weaning starting at 28 days old. Overall, these three *S.* Typhimurium candidate vaccines exhibited different levels of reactogenicity and immunogenicity in infant and young mice, dependent on the route and timing of immunization.

An initial concern was that these live-attenuated strains would be neutralized or killed by antimicrobial peptides or other components in milk, which could interfere with the activation of *Salmonella*-specific immune responses. Maternal milk contains a variety of acellular (antimicrobial peptides, cytokines, immunoglobulins) and cellular (myeloid and lymphoid cells) components that protect the infant from infection [10,39]. However, these factors would also diminish the survival and colonization of a live-attenuated vaccine. The use of luminescent versions of the candidate vaccine strains allowed us to assess dissemination and clearance in real time with a small number of mice. WT NTS strains have been detected for several days by a similar method in mice and chickens [40,41]. The luminescence kinetics were similar in infants and adults, suggesting that the vaccine strains were not killed soon after delivery in infant mice. The higher levels of luminescence observed in infants may be due to them receiving equivalent CFU of vaccine strains and, therefore, more bioluminescent bacteria per ROI. These data suggested that maternal milk did not neutralize the bacteria and that oral immunization could be effective in infant mice. These data support those of Shi et al. [19], who showed that neonatal mice orally immunized with a live-vector *S.* Typhimurium vaccine had robust bacterial loads in the intestine 3 and 7 days post-immunization and subsequent dissemination to lymphoid organs, suggesting that the inoculum was able to survive in maternal milk.

The modest-to-excellent protection provided by intranasal compared to peroral delivery could be due to vaccine stability in the absence of gastric acid or interference from immune factors ingested with maternal milk. Subunit and bacterial-like particle vaccines against *Yersinia enterocolitica* and *Shigella flexneri* have shown significant VE when delivered to adult and infant mice via the intranasal route [42,43]. The improved VE observed when some doses were delivered post-weaning on the extended schedule or in 28-day-old mice may reflect the absence of maternal milk interference and age-associated increase in immune function.

The work presented here also demonstrates that the route of immunization, age of the mice, and timing of vaccine doses, as well as the reactogenicity of the vaccines, all contribute to vaccine efficacy in infants. We generally observed that the more mutations included in the vaccine, the lower the vaccine efficacy. We previously observed vaccinemia for another CVD 1921-derived vaccine strain (CVD 1921 ∆*pipA*), suggesting that CVD 1921 may be too reactogenic [23]. While CVD 1921 ∆*htrA* and CVD 1926 were safe in infants, they elicited poor vaccine efficacy following peroral immunization of infant mice. One caveat about the challenge model that was used is that the same dose of *S.* Typhimurium was used for both adult and infant mice. It is possible that in infants, the challenge dose (which was based on the LD_50_ for adult mice) was too high and overwhelmed any potential protective effect elicited by CVD 1921 ∆*htrA* and CVD 1926.

The humoral and mucosal anti-COPS antibody responses were similar for each of the vaccines, regardless of the timing between vaccine doses or the age of immunization. While we only assessed anti-COPS titers, it is possible that antibodies to other NTS antigens may be important for clearance of infection. It is possible that other *Salmonella*-specific immune responses are not adequately stimulated by either CVD 1921 ∆*htrA* or CVD 1926. Differences in vaccine-induced antibody functionality and the ability to mediate phagocytosis and bactericidal activity may also contribute to the lack of correlation between anti-COPS antibody titers and VEs. Antibody-dependent opsonophagocytosis and complement-mediated bactericidal activity are thought to be important mechanisms for clearance or control of NTS infections and may play a role in limiting infection in our model [44,45]. Future studies could investigate differences in T-cell-mediated immunity, given the association of T-cell subsets in the incidence and severity of *Salmonella* infections in humans [46]. Additionally, there may be differences between the vaccines in terms of their ability to colonize lymphoid tissues. Benoun et al. [47] showed that an *S.* Typhimurium ∆*aroA* vaccine was able to colonize lymphoid tissues of C57BL/6 mice for 5 weeks following intravenous immunization. This vaccine elicited robust T-cell memory responses. The persistence of vaccine antigens in immune priming tissue is expected to generate robust immunity that would ultimately result in protection. We have shown that CVD 1926 elicited robust antibody and T-cell responses in adult mice [26]. However, CVD 1926, as well as CVD 1921 ∆*htrA*, viability might have been compromised during passage through the infant gut, limiting their access to lymphoid tissues and their capacity to elicit immune responses sufficiently robust to withstand a lethal challenge.

Overall, we have evaluated three live-attenuated vaccines that provide varying levels of protection in infant models of NTS infection. We determined the optimal immunization route for 14-day-old mice to be intranasal, with moderate and excellent VE for CVD 1921 ∆*htrA* (63%) and CVD 1921 (80%), respectively. Our data show that even the highly reactogenic vaccine (CVD 1921) was not able to elicit protection against lethal challenge to 14-day-old mice when administered orally at 1-week intervals. However, protection was conferred when the vaccine was administered at 2-week intervals. Additional studies should be performed to further elucidate whether there are components in the milk or the infant gut that can neutralize these live-attenuated vaccines or whether age-associated improvements in immune function account for the superior protection observed for the 2-week immunization regimen. Our superior protection data obtained for intranasal immunization suggest that one should be cautious interpreting data from animal models that use intranasal immunization, as these can achieve excellent immune responses and VE but may not reflect the conditions that these vaccines will face when administered orally.

## 5. Patents

Two patents describe these vaccines: US patent 9,050,283 and US patent 9,011,871; Inventors: Myron M. Levine, James Galen, Raphael Simon and Sharon Tennant.

## Figures and Tables

**Table 1:** *Salmonella* strains and plasmids used in this study.

Strain or Plasmid	Characteristics *	Reference
*S.* Typhimurium strain I77	Clinical isolate, sequence type 19	[29,30,31]
CVD 1921	I77 ∆*guaBA* ∆*clpP*	[22]
CVD 1921 ∆*htrA*	I77 Δ*guaBA* Δ*clpP* Δ*htrA*	This work
CVD 1926	I77 Δ*guaBA* Δ*clpP* Δ*pipA* Δ*htrA*	[23]
CVD 1925 (pSEC10-*wzzB*)	I77 ∆*guaBA* ∆*clpP ∆fliD ∆fljB* (pSEC10-*wzzB*)	[22,32]
pKD13	Kan^R^ template plasmid	[33]
pKD46	Amp^R^, expresses red recombinase	[33]
pCP20	Amp^R^ Cm^R^, FLP synthesis	[33]
pCM17	*ori*101 *luxCDABE aph hok-sok par parA rrnB*	[34]

* Amp, ampicillin; Cm, chloramphenicol.

**Table 2:** Live-attenuated *S.* Typhimurium vaccine efficacy (VE) at different dosing intervals in adult mice.

Vaccine Strain	Immunization Schedule					
	1-Month Interval			1-Week Interval		
	Mortality	VE	*p*-Value *	Mortality	VE	*p*-Value *
CVD 1921	6/28	79%	<0.0001	0/15	93%	<0.0001
CVD 1921 ∆*htrA*	14/30	53%	<0.0001	5/15	64%	0.017
CVD 1926	14/29	52%	<0.0001	5/15	64%	0.017
PBS	30/30	-	-	14/15	-	-

* Fisher’s exact test, two-tailed.

**Table 3:** *S.* Typhimurium live-attenuated vaccine efficacy (VE) via intranasal (i.n.) delivery in infant mice.

Vaccine Group	Mortality	VE	*p*-Value *
CVD 1921	3/15	80%	<0.0001
CVD 1921 ∆*htrA*	6/16	63%	0.0002
CVD 1926	11/16	31%	0.0434
PBS	15/15	-	-

* Fisher’s exact test, two-tailed. Mortality is represented as dead mice/total mice challenged.

**Table 4:** Vaccine efficacy elicited by *S.* Typhimurium vaccines in infant and young BALB/c mice.

Vaccine Group	Age and Immunization Schedule								
	14-Day-Old						28-Day-Old		
	1-Week Interval			2-Week Interval			2-Week Interval		
	Mortality	VE	*p*-Value *	Mortality	VE	*p*-Value *	Mortality	VE	*p*-Value *
CVD 1921	8/15	34%	0.1351	3/17	79%	0.0004	2/16	83%	0.010
CVD 1921 ∆*htrA*	12/15	1.5%	1.0000	10/17	29%	0.2587	6/14	43%	0.1349
CVD 1926	9/15	26%	0.2524	10/16	24%	0.2587	5/16	58%	0.0320
PBS	13/16	-		14/17	-		12/16	-	

* Fisher’s exact test, two-tailed.

## Data Availability

All data are contained within this article.
